# Supplementary material for: Validity and reliability of an app-based medical device to empower individuals in evaluating their physical capacities
Source: PLoS One. 2023 Aug 10;18(8):e0289874. doi: 10.1371/journal.pone.0289874 (PMC10414674; doi:10.1371/journal.pone.0289874)
Supplement: S1 Table — (PDF) [file pone.0289874.s001.pdf]

S1 Table. Summary of the results stratified by age (participants <30 years old vs participants ≥ 30 years old).

Variables	< 30 years old	≥ 30 years old
N participants	26	26
Mean age (years)	25 (±2.0)	43 (±10.7)
6MWT mean performance (meters)	685.78 (±63.3)	700.77 (±92.2)
STS mean performance (repetitions)	21.0 (±5.4)	22.4 (±6.0)
6MWT Validity (Pearson correlation)	0.78 (0.70; 0.85)	0.86 (0.81; 0.91)
STS Validity (Pearson correlation)	0.99 (0.99; 0.99)	0.98 (0.97; 0.99)
6MWT Relative measurement error (%)	4.26	4.69
STS Relative measurement error (%)	1.23	2.01
6MWT Concordance correlation coefficient (CCC)	0.77 (0.67; 0.85)	0.86 (0.78; 0.91)
STS Concordance correlation coefficient (CCC)	0.99 (0.98 ; 0.99)	0.98 (0.97 ; 0.99)
6MWT Reliability (ICC2,1)	0.66 (0.46; 0.81)	0.84 (0.72; 0.92)
STS Reliability (ICC2,1)	0.78 (0.62; 0.88)	0.70 (0.50; 0.85)
6MWT Standard error measurement (meters)	37.80 (26.83; 48.77)	34.47 (23.10; 43.87)
STS Standard error measurement (repetitions)	2.46 (2.04; 4.20)	2.94 (2.03; 3.84)
6MWT Coefficient of variation (%)	8.97	10.51
STS Coefficient of variation (%)	26.02	21.50
6MWT Minimal detectable change (meters)	104.78	95.35
6MWT Minimal detectable change (%)	15.09	15.08
STS Minimal detectable change (repetitions)	6.82	8.15
STS Minimal detectable change (%)	32.81	35.98
