# Supplementary material for: Validity and reliability of an app-based medical device to empower individuals in evaluating their physical capacities
Source: PLoS One. 2023 Aug 10;18(8):e0289874. doi: 10.1371/journal.pone.0289874 (PMC10414674; doi:10.1371/journal.pone.0289874)
Supplement: S2 Table — (PDF) [file pone.0289874.s002.pdf]

S2 Table. Summary of the results stratified by sex (men vs women).

**Variables**	**Men**	**Women**
N participants	23	30
Mean age (years)	32.91 (±9.7)	33.39 (±11.9)
6MWT mean performance (meters)	709.74 (±91.8)	684.62 (±62.4)
STS mean performance (repetitions)	22.22 (±5.4)	22.00 (±6.4)
6MWT Validity (Pearson correlation)	0.90 (0.70; 0.85)	0.88 (0.82; 0.92)
STS Validity (Pearson correlation)	0.98 (0.97; 0.99)	0.99 (0.99; 0.99)
6MWT Relative measurement error (%)	3.88	4.80
STS Relative measurement error (%)	1.91	1.59
6MWT Concordance correlation coefficient (CCC)	0.90 (0.84; 0.94)	0.73 (0.61; 0.81)
STS Concordance correlation coefficient (CCC)	0.98 (0.97 ; 0.99)	0.99 (0.98 ; 0.99)
6MWT Reliability (ICC2,1)	0.75 (0.55; 0.88)	0.83 (0.701; 0.91)
STS Reliability (ICC2,1)	0.58 (0.33; 0.78)	0.89 (0.80; 0.94)
6MWT Standard error measurement (meters)	43.24 (29.26; 57.22)	26.77 (20.36; 33.18)
STS Standard error measurement (repetitions)	3.10 (2.13; 4.07)	2.16 (1.64; 2.67)
6MWT Coefficient of variation (%)	11.17	8.91
STS Coefficient of variation (%)	21.59	28.52
6MWT Minimal detectable change (meters)	119.74	74.20
6MWT Minimal detectable change (%)	16.61	10.72
STS Minimal detectable change (repetitions)	8.59	5.99
STS Minimal detectable change (%)	38.88	27.60
